# Supplementary material for: DT-PICS: An Efficient and Cost-Effective SNP Selection Method for the Germplasm Identification of Arabidopsis
Source: Int J Mol Sci. 2023 May 14;24(10):8742. doi: 10.3390/ijms24108742 (PMC10218072; doi:10.3390/ijms24108742)
Supplement: Supplementary file 1 [file ijms-24-08742-s001.zip › Document.S1.pdf]

Document S1: The Rscript for DT-PICS method.

Stage 1: preliminary screening of SNP markers.

```
# data is the raw data, k is the number of list head, x is for's times
Cutpic<-function(data,k,x){

  mypaste<-function(x){
    G<-paste(x,collapse = "")
    G
  }

  datatest<-NULL
  datatest2<-NULL
  picsnps<-NULL
  G<-NULL
  datatest<-as.matrix(data)
  datatest2<-as.matrix(data)
  ccol<-(ncol(datatest)-k)
  snpsite<-1:nrow(datatest)
  snp<-NULL
  list_A<-list(c(1:ccol))
  length_1<-NULL
  length_1[1]<-1
  count_1<-NULL

  # split the dataset based on overall PIC value now
  for(j in 1:x){

    k1<-0
    for (i_2 in 1:length_1[j]) {
      k2<-PICnow(datatest[,c(1:k,k+unlist(list_A[i_2]))],k)
      k1<-k2+k1
    }
    if(length(which(k1==0))!=0)
    {snpsite<-snpsite[-which(k1==0)]
    datatest<-datatest[-which(k1==0),]
    k1<-k1[-which(k1==0)]
    }
    picsnps[j]<-sample(c(order(k1,decreasing=TRUE)[1:ceiling(length(k1)*0.1)]),1)
    snp[j]<-snpsite[picsnps[j]]
```

```
        if(length(snp)>1)
            {G<-apply(datatest2[snp,-c(1:k)],2,mypaste)}
        else
            {G<-datatest2[snp,-c(1:k)]}

        table_A<-as.data.frame(table(G))
        char_1<-as.character(table_A[,1])
        length_1[j+1]<-length(char_1)
        count_1[j]<-length(which(table_A[,2]%in%1))
        if(length_1[j+1]==ccol)(break)

        list_A<-NULL
        for (i_1 in 1:length_1[j+1]) {
            list_A[i_1]<-list(which(G%in%char_1[i_1]))
            }
    }
    return(snp)
}

# calculate the PIC now for split dataset
PICnow<-function(syan6,k){
    G<-NULL
    kpicgsnp<-syan6
    kpicgsnp<-as.matrix(kpicgsnp)
    ccol<-(ncol(kpicgsnp)-k)
    rrow<-nrow(kpicgsnp)
    rrlt<-NULL
    for(i in 1:rrow){
        G<-kpicgsnp[i,-c(1:k)]

        pictable<-table(G)
        pictable2<-as.numeric(pictable)
        m=1
        for(n in 1:length(pictable2)){
            m=m-(pictable2[n]/ccol)**2
        }
        rrlt[i]<-m
    }
    rrlt
}

# used the function
nchrz2<-read.table("nchrx.txt",stringsAsFactors = F)
Cutpic(nchrz2,5,150)
```

Stage 2: Redundant SNP deletion.

```
#data1 is the raw data, samp is for's times, k is the number of list head
#ans is the un-simplified SNP set, snpsite is which the snpsite in data1
Newfinish<-function(data1,samp,ans,k,snpsite){

snpss<-list(NULL)
gmax<-list(NULL)
list1<-list(NULL)
ge<-NULL
SNPS<-data1[which((as.character(data1[,snpsite]))%in%unique(ans)),]

# ensure the SNP is redundant or nor
  sapply(1:samp,function(j){
  T<-sample(ans,length(ans))
  i=1
    repeat {
      p<-length(T)
      t<-T[-i]
      rlt<-identifiy(SNPS,t,k,snpsite)
      if(rlt==(length(SNPS[1,])-k)){T<-t;rlt=0;}else(i=i+1)
      if(p==(i-1))(break)
      print(i)
    }
  snpss[j]<<-list(as.character(T))
  ge[j]<<-length(as.character(T))
})
gmax[1]<-list(min(ge))
gmax[2]<-list(snpss[which.min(ge)])
list1<-list(snpss,ge,gmax)
return(list1)
}

# used the function
Newfinish(data1,500,snpset,6,1)
```
