# Supplementary material for: DT-PICS: An Efficient and Cost-Effective SNP Selection Method for the Germplasm Identification of Arabidopsis
Source: Int J Mol Sci. 2023 May 14;24(10):8742. doi: 10.3390/ijms24108742 (PMC10218072; doi:10.3390/ijms24108742)
Supplement: Supplementary file 1 [file ijms-24-08742-s001.zip › Figure S7.pdf]

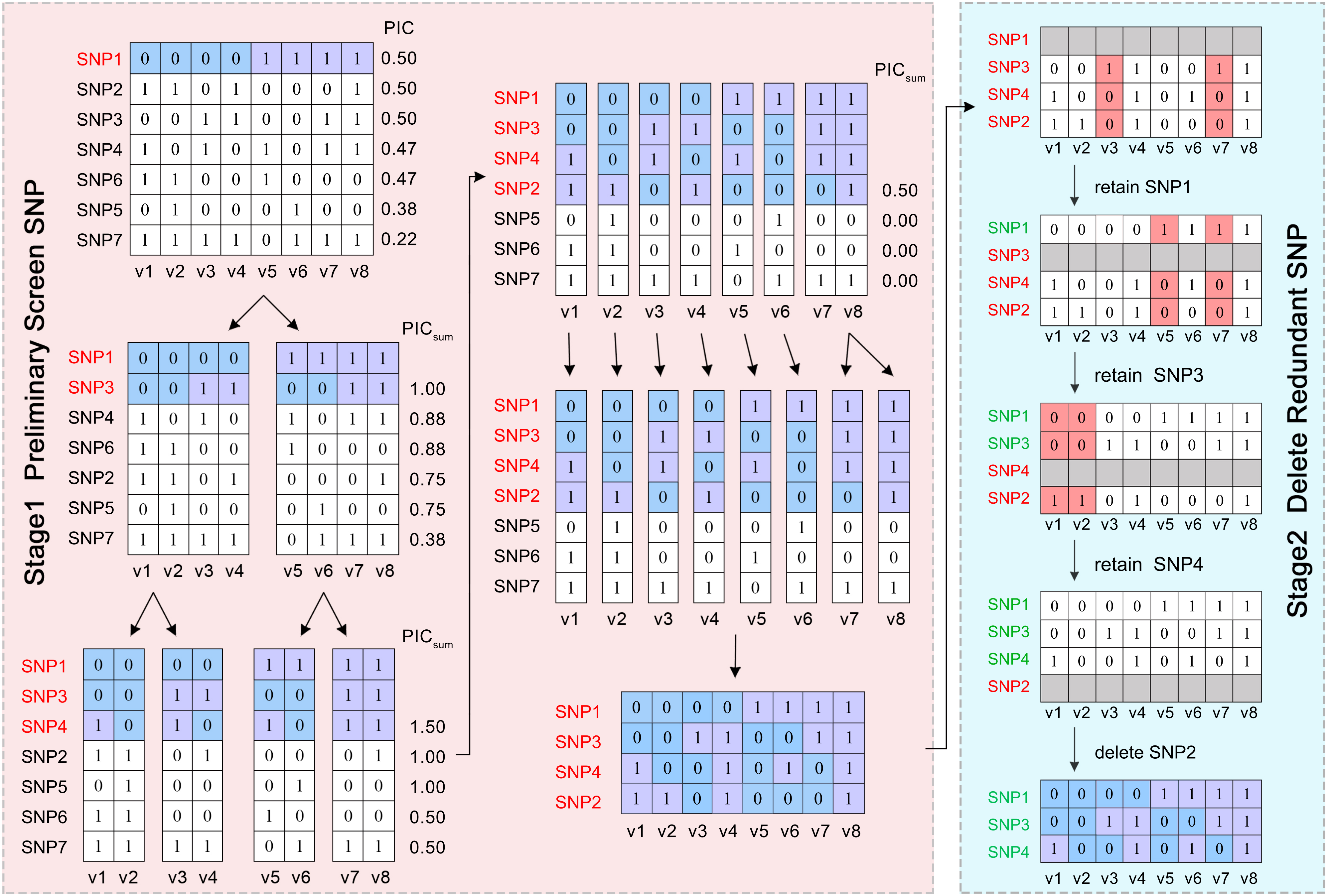
Stage1 Preliminary Screen SNP
Stage2 Delete Redundant SNP
PIC
SNP1 0.50
SNP2 0.50
SNP3 0.50
SNP4 0.47
SNP6 0.47
SNP5 0.38
SNP7 0.22
v1 v2 v3 v4 v5 v6 v7 v8
PIC$_{sum}$
SNP1
SNP3 1.00
SNP4 0.88
SNP6 0.88
SNP2 0.75
SNP5 0.75
SNP7 0.38
PIC$_{sum}$
SNP1
SNP3
SNP4 1.50
SNP2 1.00
SNP5 1.00
SNP6 0.50
SNP7 0.50
PIC$_{sum}$
SNP1
SNP3
SNP4
SNP2 0.50
SNP5 0.00
SNP6 0.00
SNP7 0.00
retain SNP1
retain SNP3
retain SNP4
delete SNP2
