# Supplementary figures and images for: DT-PICS: An Efficient and Cost-Effective SNP Selection Method for the Germplasm Identification of Arabidopsis
Source: Int J Mol Sci. 2023 May 14;24(10):8742. doi: 10.3390/ijms24108742 (PMC10218072; doi:10.3390/ijms24108742)

**A**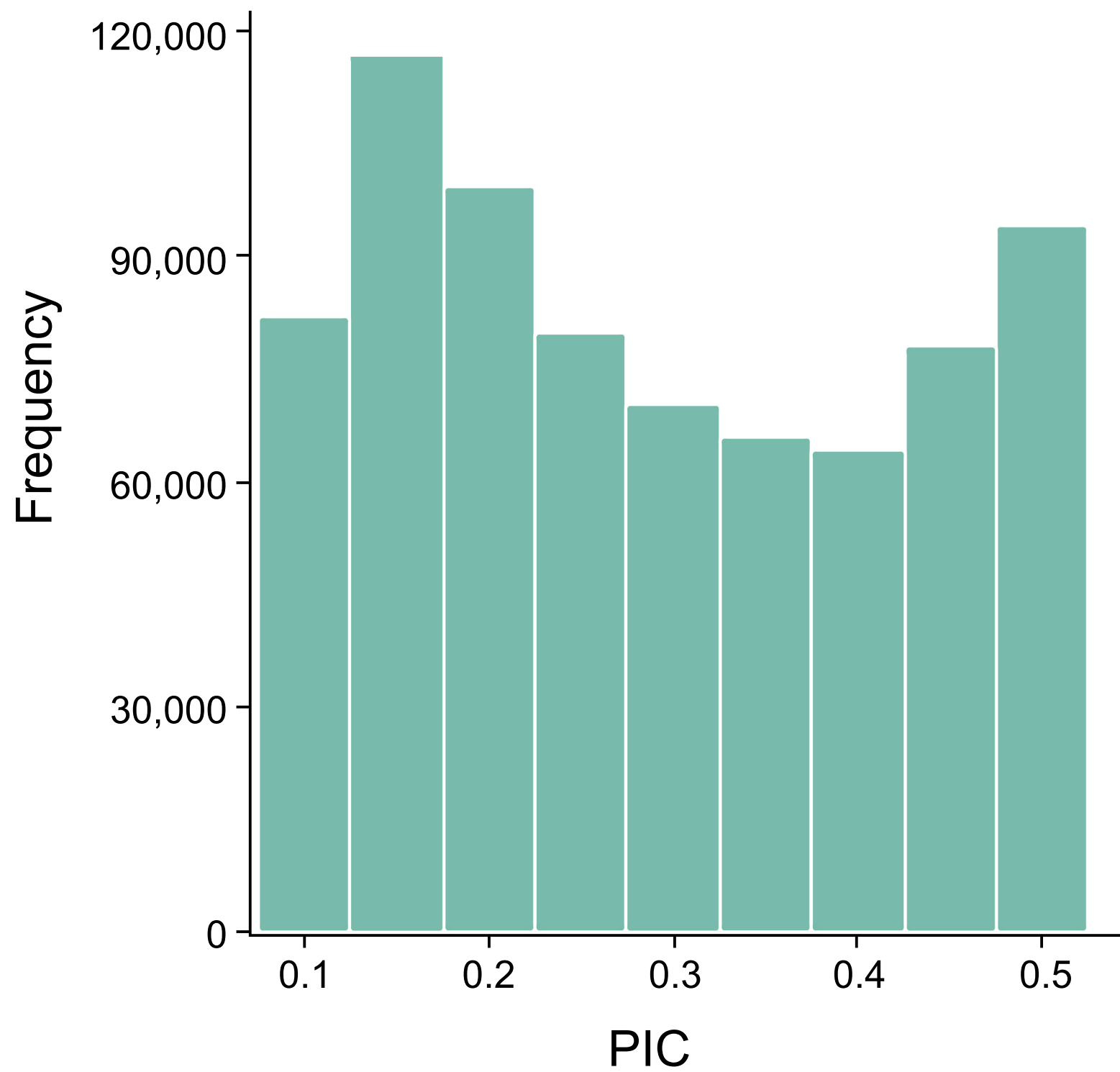**B**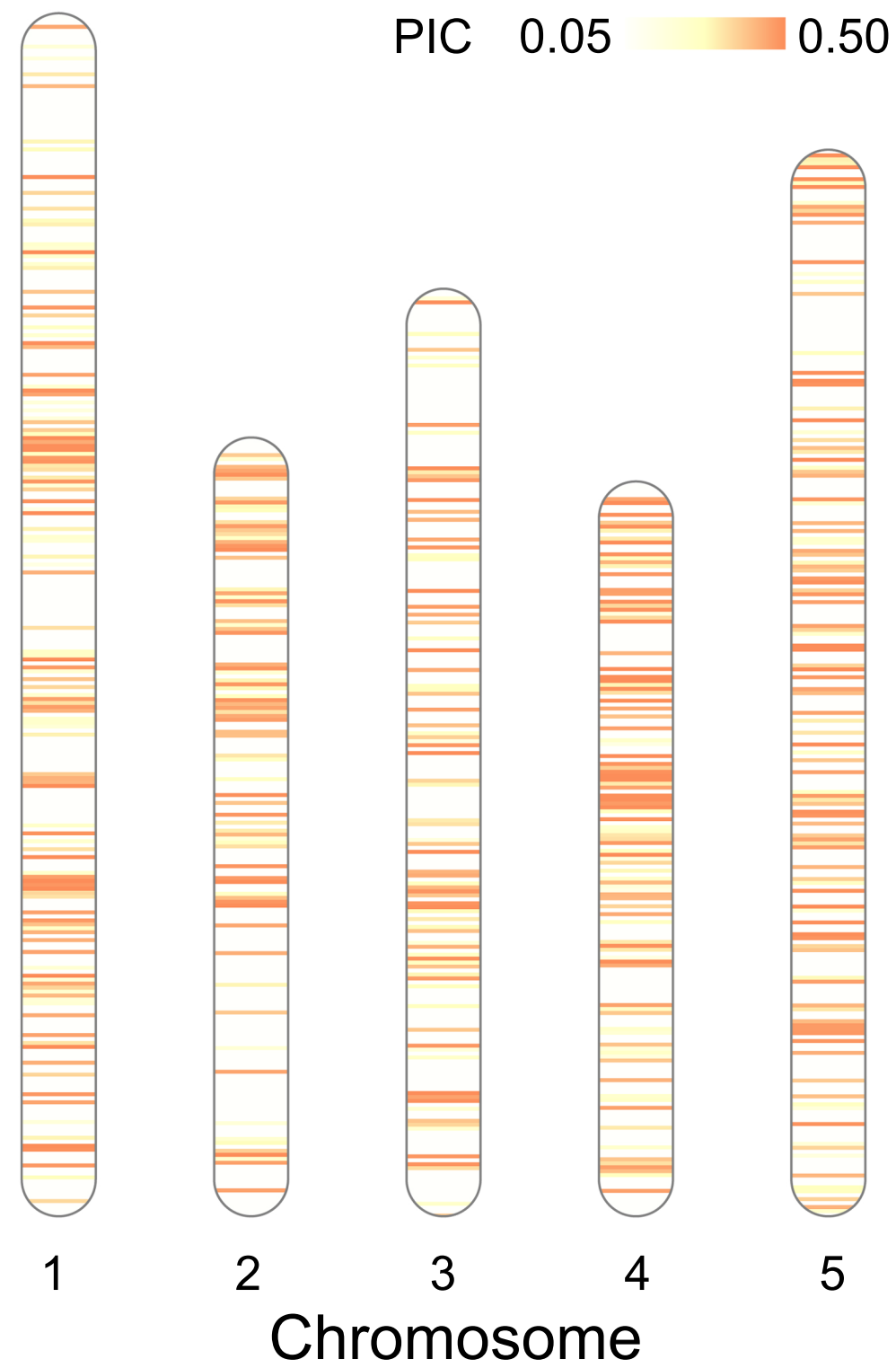

Supplement: Supplementary file 1 [file ijms-24-08742-s001.zip › Figure S1.pdf]

**A**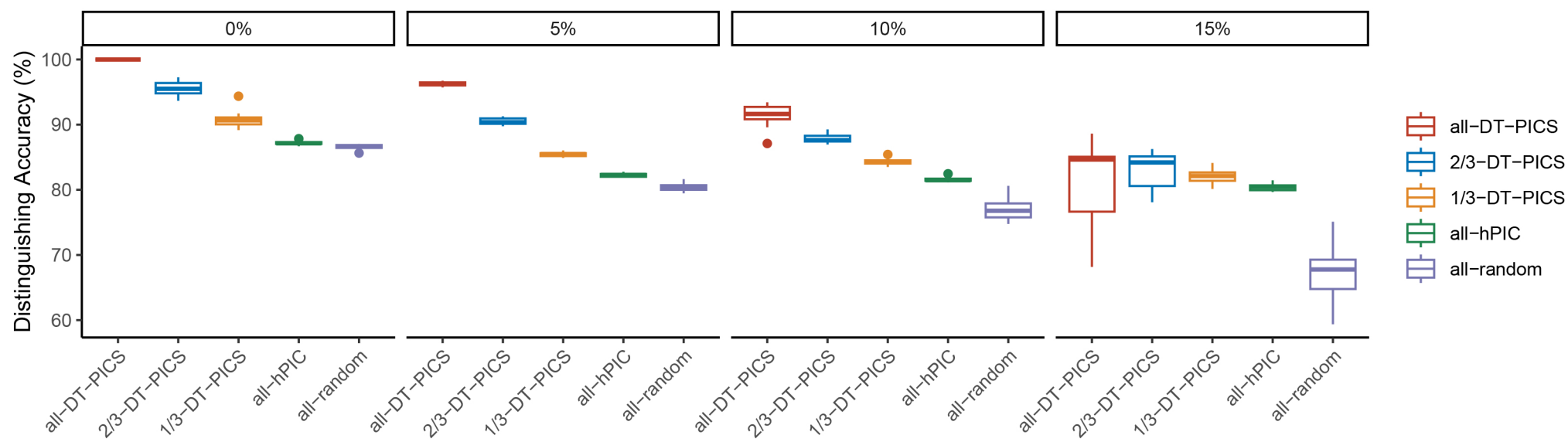**B**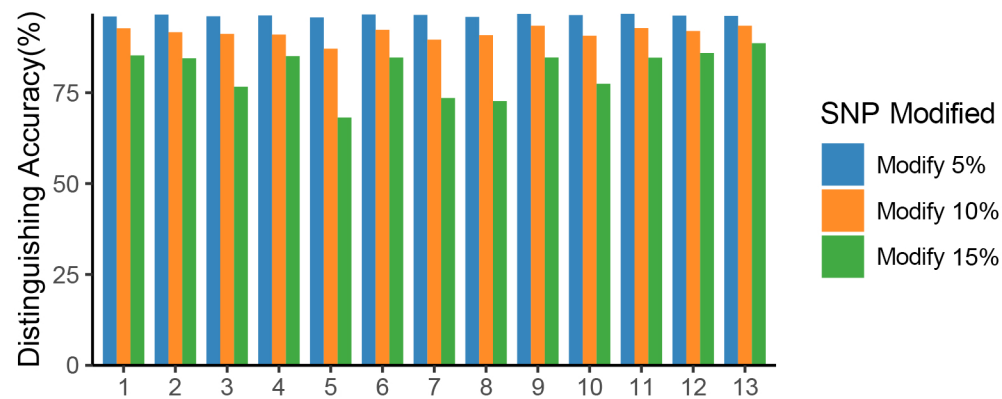**C**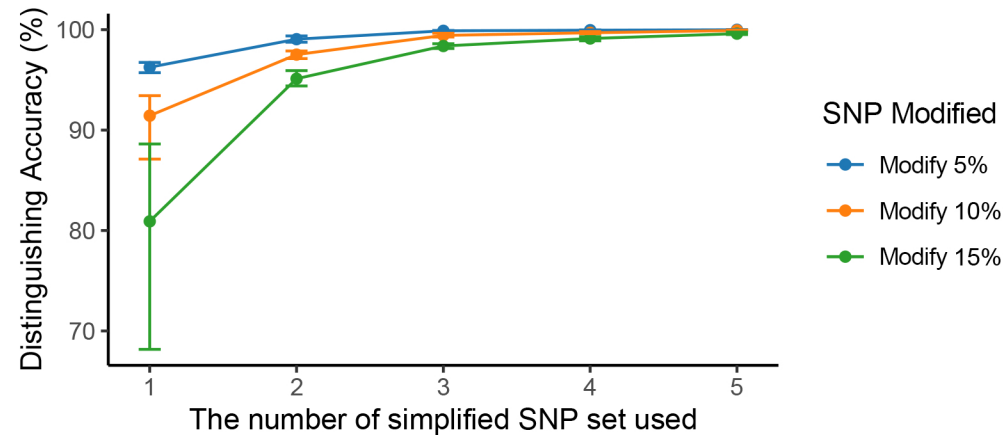

Supplement: Supplementary file 1 [file ijms-24-08742-s001.zip › Figure S2.pdf]

**A**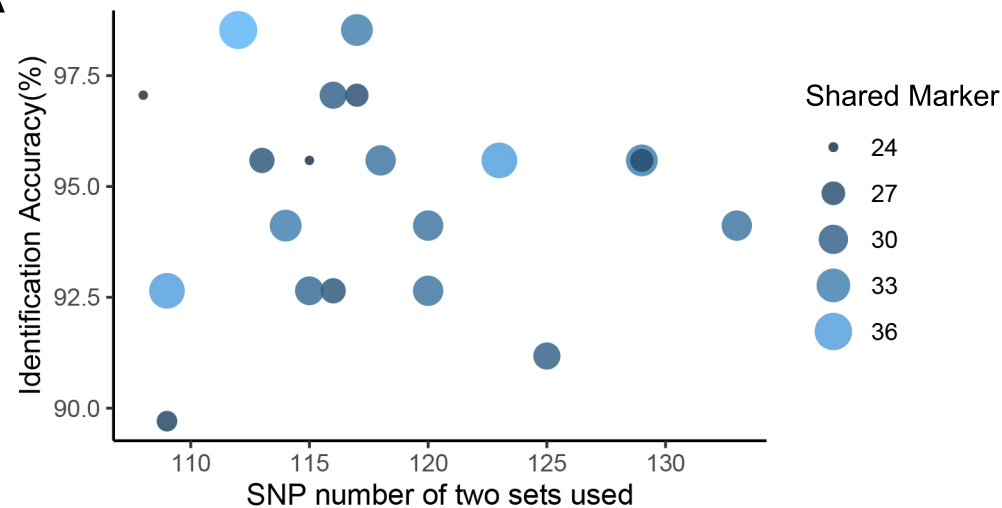**B**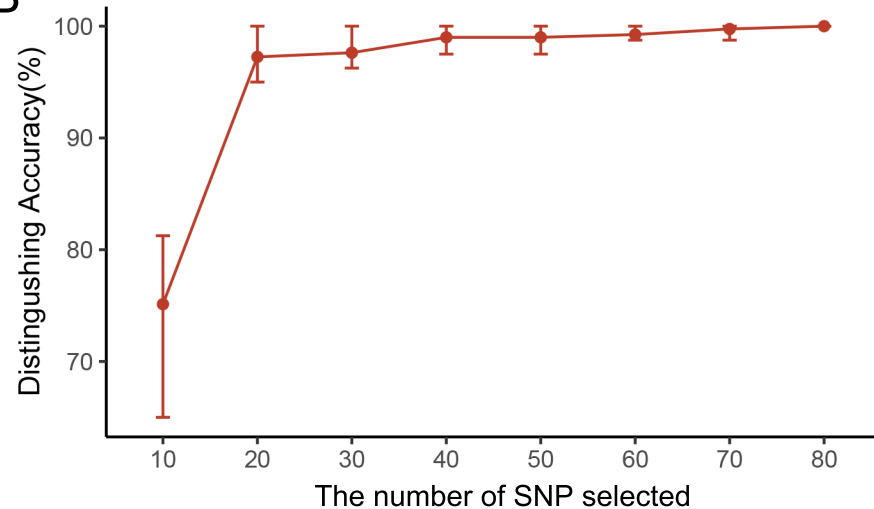**C**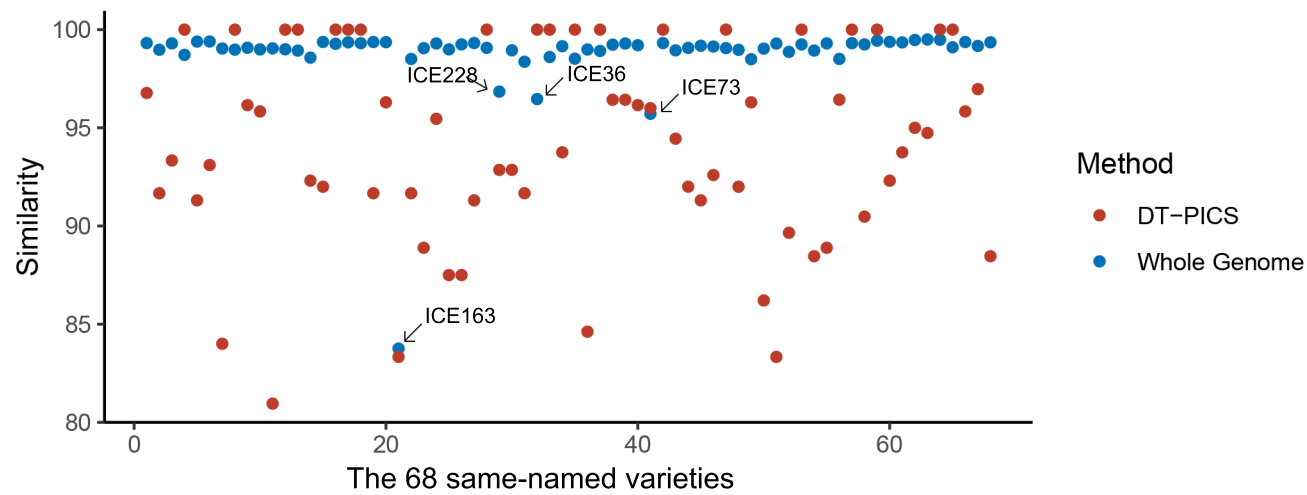

Supplement: Supplementary file 1 [file ijms-24-08742-s001.zip › Figure S3.pdf]

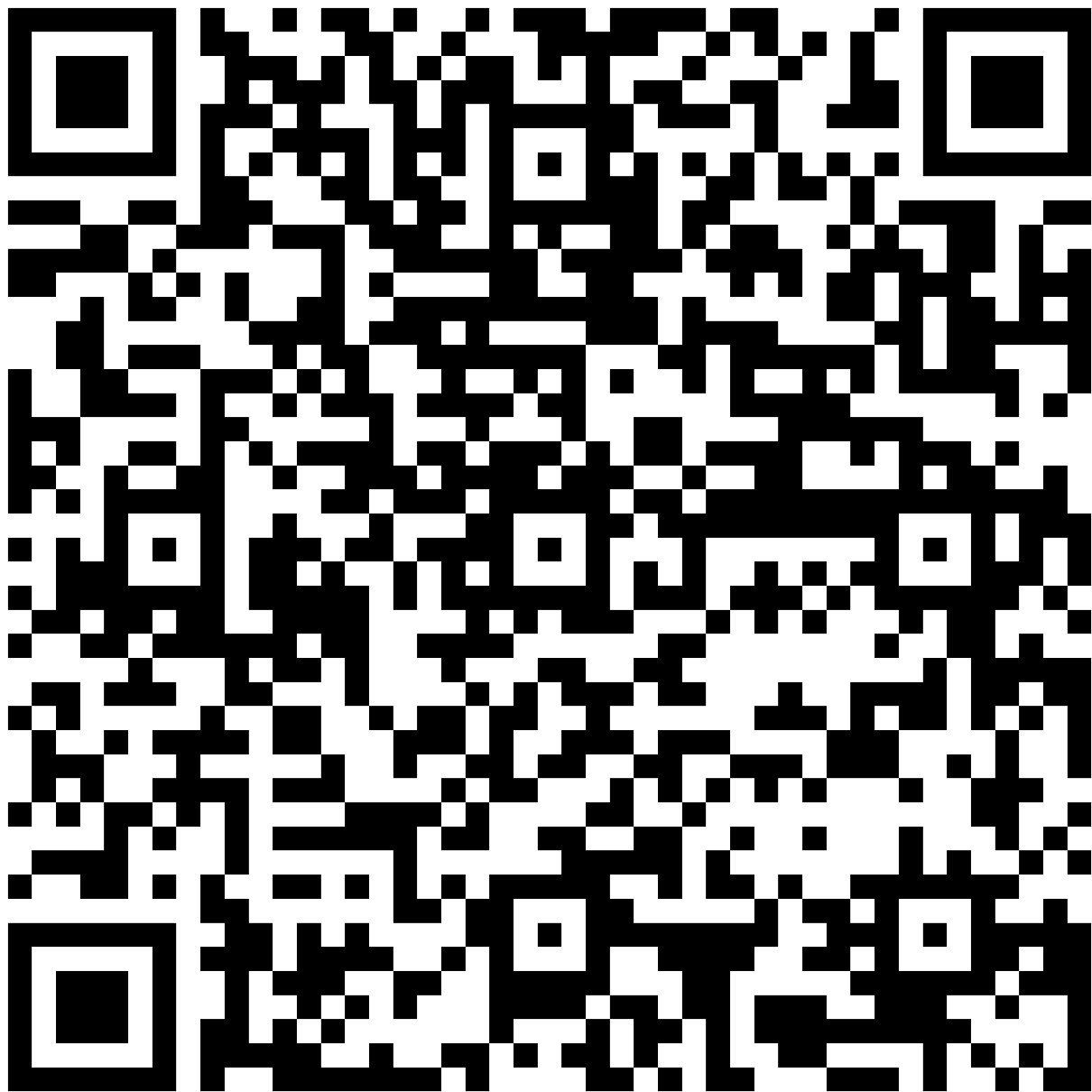

Supplement: Supplementary file 1 [file ijms-24-08742-s001.zip › Figure S4.pdf]

Train\_Data (1135)

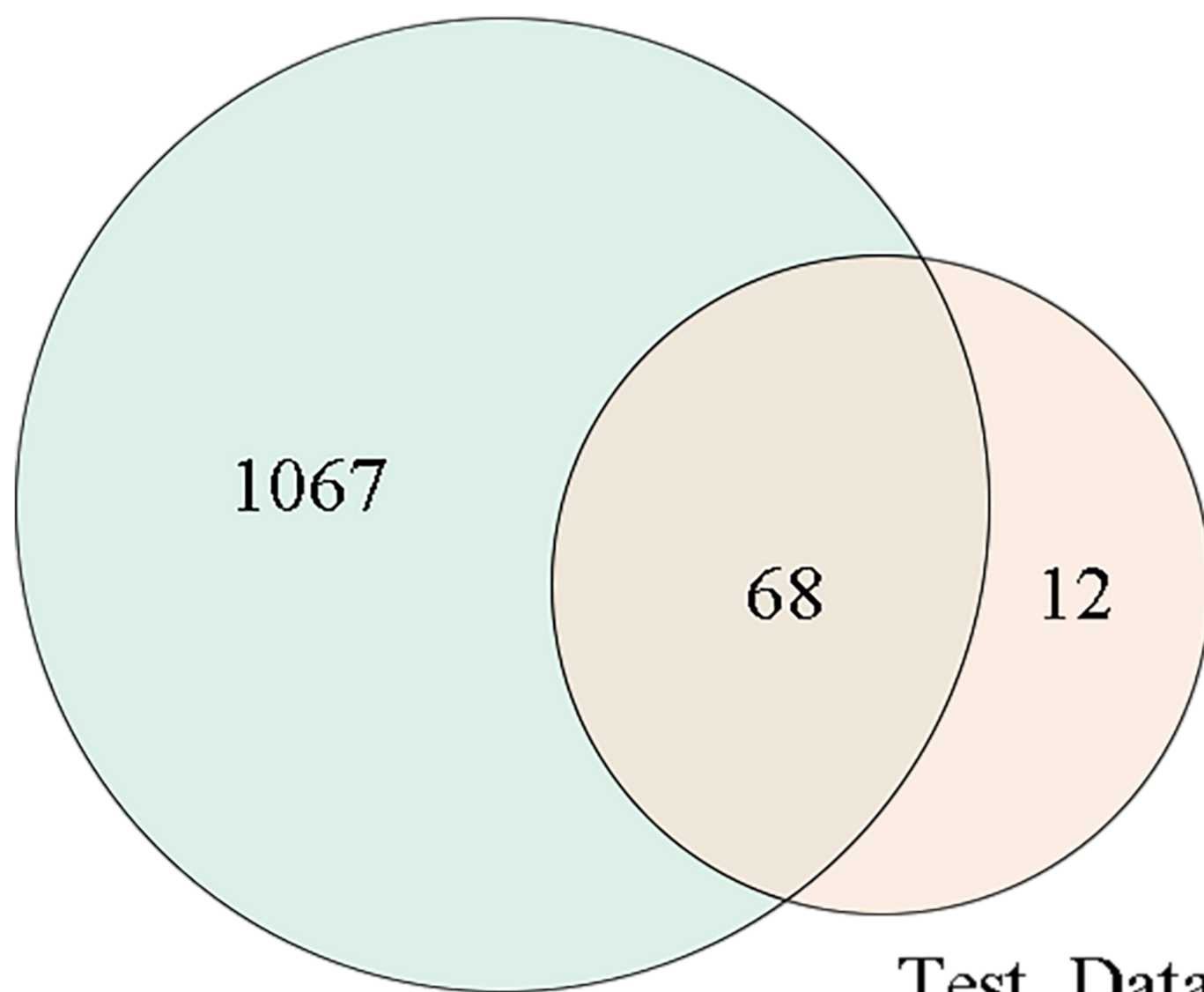

Test\_Data (80)

Supplement: Supplementary file 1 [file ijms-24-08742-s001.zip › Figure S5.pdf]

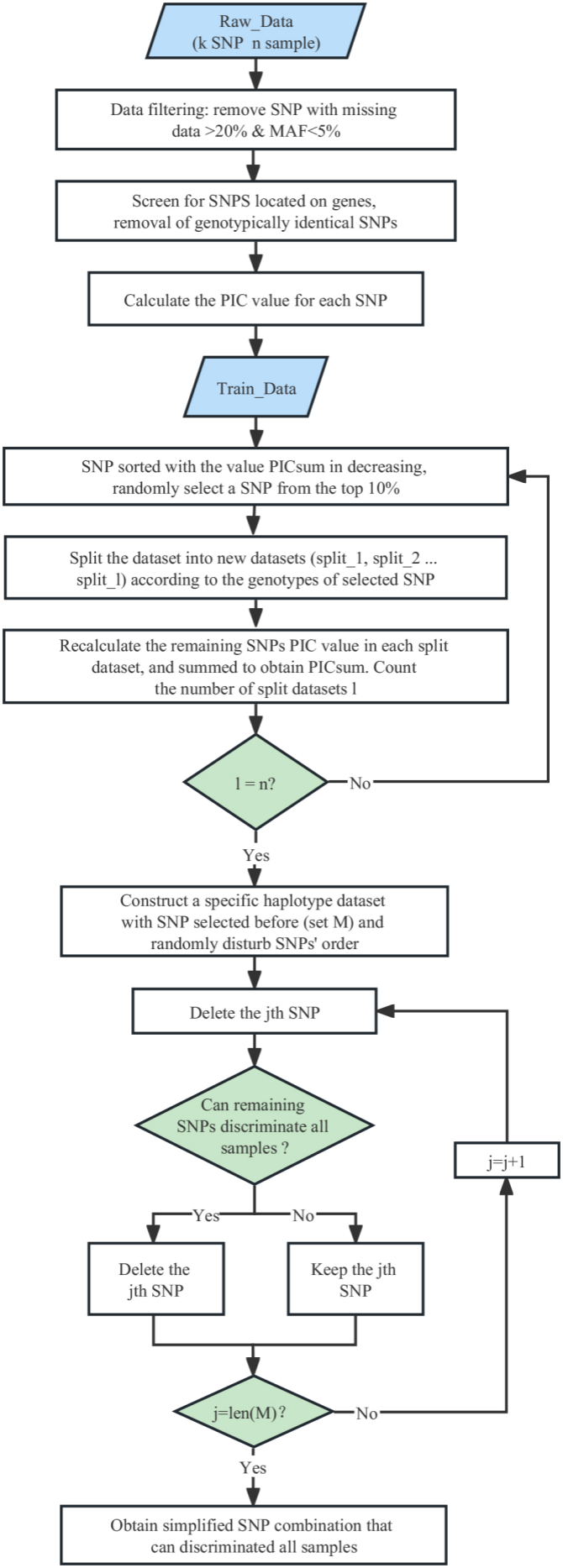

Supplement: Supplementary file 1 [file ijms-24-08742-s001.zip › Figure S6.pdf]
